# MRI guided procedure planning and 3D simulation for partial gland cryoablation of the prostate: a pilot study

**DOI:** 10.1186/s41205-020-00085-2

**Published:** 2020-11-03

**Authors:** Nicole Wake, Andrew B. Rosenkrantz, Daniel K. Sodickson, Hersh Chandarana, James S. Wysock

**Affiliations:** 1 Department of Radiology, Montefiore Medical Center, Albert Einstein College of Medicine, 111 East 210th Street, Bronx, NY 10467 USA; 2 Center for Advanced Imaging Innovation and Research (CAI2R) and Bernard and Irene Schwartz Center for Biomedical Imaging, Department of Radiology, NYU Langone Health, NYU Grossman School of Medicine, New York, NY USA; 3 Division of Urologic Oncology, Department of Urology, NYU Langone Health, NYU Grossman School of Medicine, New York, NY USA

**Keywords:** Prostate cancer, Cryotherapy, 3D planning, MRI

## Abstract

**Purpose:**

This study reports on the development of a novel 3D procedure planning technique to provide pre-ablation treatment planning for partial gland prostate cryoablation (cPGA).

**Methods:**

Twenty men scheduled for partial gland cryoablation (cPGA) underwent pre-operative image segmentation and 3D modeling of the prostatic capsule, index lesion, urethra, rectum, and neurovascular bundles based upon multi-parametric MRI data. Pre-treatment 3D planning models were designed including virtual 3D cryotherapy probes to predict and plan cryotherapy probe configuration needed to achieve confluent treatment volume. Treatment efficacy was measured with 6 month post-operative MRI, serum prostate specific antigen (PSA) at 3 and 6 months, and treatment zone biopsy results at 6 months. Outcomes from 3D planning were compared to outcomes from a series of 20 patients undergoing cPGA using traditional 2D planning techniques.

**Results:**

Forty men underwent cPGA. The median age of the cohort undergoing 3D treatment planning was 64.8 years with a median pretreatment PSA of 6.97 ng/mL. The Gleason grade group (GGG) of treated index lesions in this cohort included 1 (5%) GGG1, 11 (55%) GGG2, 7 (35%) GGG3, and 1 (5%) GGG4. Two (10%) of these treatments were post-radiation salvage therapies. The 2D treatment cohort included 20 men with a median age of 68.5 yrs., median pretreatment PSA of 6.76 ng/mL. The Gleason grade group (GGG) of treated index lesions in this cohort included 3 (15%) GGG1, 8 (40%) GGG2, 8 (40%) GGG3, 1 (5%) GGG4. Two (10%) of these treatments were post-radiation salvage therapies. 3D planning predicted the same number of cryoprobes for each group, however a greater number of cryoprobes was used in the procedure for the prospective 3D group as compared to that with 2D planning (4.10 ± 1.37 and 3.25 ± 0.44 respectively, *p* = 0.01). At 6 months post cPGA, the median PSA was 1.68 ng/mL and 2.38 ng/mL in the 3D and 2D cohorts respectively, with a larger decrease noted in the 3D cohort (75.9% reduction noted in 3D cohort and 64.8% reduction 2D cohort, *p* 0.48). In-field disease detection was 1/14 (7.1%) on surveillance biopsy in the 3D cohort and 3/14 (21.4%) in the 2D cohort, *p = 0.056)* In the 3D cohort, 6 month biopsy was not performed in 4 patients (20%) due to undetectable PSA, negative MRI, and negative MRI Axumin PET. For the group with traditional 2D planning, treatment zone biopsy was positive in 3/14 (21.4%) of the patients, *p* = 0.056.

**Conclusions:**

3D prostate cancer models derived from mpMRI data provide novel guidance for planning confluent treatment volumes for cPGA and predicted a greater number of treatment probes than traditional 2D planning methods. This study prompts further investigation into the use of 3D treatment planning techniques as the increase of partial gland ablation treatment protocols develop.

## Introduction

The utilization of multiparametric magnetic resonance imaging (mpMRI) in the diagnostic paradigm for prostate cancer has emerged as the primary imaging modality utilized to identify and characterize clinically significant prostate cancer [1–5]. Coupling mpMRI with targeted prostate biopsy using MRI ultrasound fusion increases detection of clinically significant prostate cancer and enables accurate disease localization thus opening the possibility of targeted treatment via prostate gland ablation (PGA) [3, 6, 7]. While mpMRI accurately identifies disease location, multiple studies demonstrate that it underestimates the exact tumor volume, up to 30% in some studies [8–10]. This volume underestimation results in the need to increase the amount of prostate treated in order to ensure ablation of the MR-visible tumor as well as the invisible boundaries. As an example, working from radical prostatectomy specimens, Le Nobin et al reported that a treatment margin of approximately 13 mm around image visible disease was needed to ensure adequate disease capture [11].

Prostate ablation has been reported using multiple energy sources including radiofrequency thermal ablation, vascular targeted photodynamic therapy, high intensity focused ultrasound, irreversible electroporation, as well as cryoablation [12]. Prostate cryoablation destroys prostate cancer by creating zones of ice via transperineal needles (cryoprobes). Cycling the tissue between multiple freeze and thaw cycles achieves tissue destruction via cellular membrane disruption, microthrombi and ischemia [13]. Clinical application of cryoablation for performing partial gland ablation as both primary treatment for localized prostate cancer as well as for salvage treatment following radiation therapy have been described [14–16]. Reported outcomes for prostate cryoablation demonstrate positive biopsy rates from 12% to 38% [17–20].

During cryoablation, the probes are placed using two-dimensional (2D) image guidance for localization of prostate tumor, and the lesion is targeted visually (aka with cognitive fusion). The development of three-dimensional (3D) treatment volumes of ice at − 40 °C ensures tissue destruction [15]. Standard of care cryoablation is achieved by placing cryoprobes into the tissue under 2D ultrasound guidance. The tumor volume and margins are estimated. Ultimately, the success of partial gland prostate cryoablation (cPGA) depends upon the development of a 3D ablation volume that entirely encompasses the tumor and its margin within a zone of at least − 40 °C. Utilizing the correct number of cryoprobes in the correct spatial orientation is necessary to achieve this goal [21].

In order to overcome the shortcomings of 2D imaging techniques for pre-operative planning, 3D surgical planning has been applied in areas such as craniomaxillofacial surgery [22], orthopedic surgery [23], liver cryotherapy [24], and radiofrequency ablation [25–28]. However, with respect to prostate cancer cryoablation, at the time of this study development, commercial software relies upon 2D images and was developed for whole gland ablation, and no commercial tools are available to guide treatment for cPGA in 3D.

To address the inadequacies inherent to 2D mapping techniques, this study reports on the development of a novel 3D procedure planning technique to provide pre-ablation treatment planning for cPGA. Patient-specific 3D models based upon mpMRI are created and the cPGA procedure is simulated using virtual 3D cryoprobes. Prior to cPGA, virtual 3D planning is utilized to confirm the required number and placement of cryoprobes to achieve confluent treatment volume for each unique lesion and margin. Optimization of the treatment plan in 3D by placing a predefined number of cryotherapy probes to best cover the lesion with the estimated − 40 °C isotherm surface is expected to save time during the surgical procedure and to ultimately to help improve outcomes following cryotherapy for prostate cancer.

## Methods

Consecutive men were offered inclusion into this study after enrollment in a prospective registry evaluating oncologic and functional outcomes following cryoablation [29]. Briefly, men included in this registry were diagnosed with either clinically localized prostate cancer or radiorecurrent prostate cancer. Pre-operative mpMRI was performed at 3 T (Skyra, Siemens, Erlangen, Germany) and included a 3D turbo spin-echo T2-weighted imaging sequence (i.e. SPACE) with a 0.6 mm × 0.66 mm × 1.00 mm, diffusion-weighted imaging (DWI), and dynamic contrast-enhanced (DCE) images. Ten individual radiologists full time academic radiologists with extensive training and various levels of experience interpreted the images. All men were found to have MRI that demonstrated a lesion with PI RADS v2 score ≥ 3 on prebiopsy evaluation.

Diagnostic biopsy was performed using transrectal MRI-ultrasound fusion on the Artemis™ platform (technique previously described) [30]. Cryoablation of the prostate is an FDA approved treatment for prostate cancer and was offered as a treatment option for men as part of a prospective registry evaluating the outcomes of this novel treatment strategy. Men who agreed to proceed with cPGA also agreed to surveillance MRI and prostate biopsy. Men with biopsy proven local recurrence following radiation therapy were also considered for inclusion in the cyroablation registry. The impetus for exploring the role for partial gland ablation for prostate cancer is beyond the scope of this manuscript [31]. However, men selected this treatment option based upon the potential to attain oncologic outcomes comparable to whole gland treatment while minimizing impact on benign prostate tissue and surrounding organs such as the neurovascular bundle, urethra, and bladder, In addition to primary treatment, cPGA offers a potential treatment for men with local recurrence following radiation treatment. Treatment options for these men are limited and carry higher side effect profiles compared to de-novo invasive treatment options [32]. Ultimately, men enrolled in the registry with MRI-visible, biopsy proven prostate cancer (PI-RADS v2 score ≥ 3) scheduled to undergo cPGA (*n* = 20) were enrolled in our Institutional Review Board approved prospective study investigating advanced methods of data visualization for patients with prostate cancer. Patient-specific 3D prostate cancer models were developed as described below. An additional comparison group (n = 20) composed of men undergoing cPGA using 2D planning techniques was retrospectively enrolled from the cryoablation registry were evaluated. The patient demographics for the 2D and 3D planning groups are shown in Table 1. Two patients from each cohort (total of 4 men, 10%) were treated with cryoablation as a salvage treatment following radiation therapy. Statistical analyses were performed in Matlab R2017a (The Mathworks Inc., Natick, MA). Continuous variables were compared using a t-test and categorical variables using the Mann-Whitney U-test.

**Table 1:** Patient demographic information

	3D Planning	2D Planning	*P*-value
Age (years)			
Mean	65	66	0.71
Range	50–73	52–80	
PSA (ng/mL)	6.78 ± 4.02	6.42 ± 3.80	0.66
PI-RADs			0.09
Score = 2	*n* = 0	*n* = 1	
Score = 3	*n* = 8	*n* = 11	
Score = 4	*n* = 7	*n* = 7	
Score = 5	*n* = 5	*n* = 1	
Lesion volume (cm^3^)	1.03 ± 1.61	0.38 ± 0.32	0.14
Gleason Score			0.91
3 + 3	*n* = 1	*n* = 3	
3 + 4	*n* = 11	*n* = 8	
4 + 3	*n* = 6	*n* = 7	
4 + 4	*n* = 1	*n* = 2	
4 + 5	*n* = 1	*n* = 0	

### Patient-specific 3D prostate cancer models

**Fig. 1:**
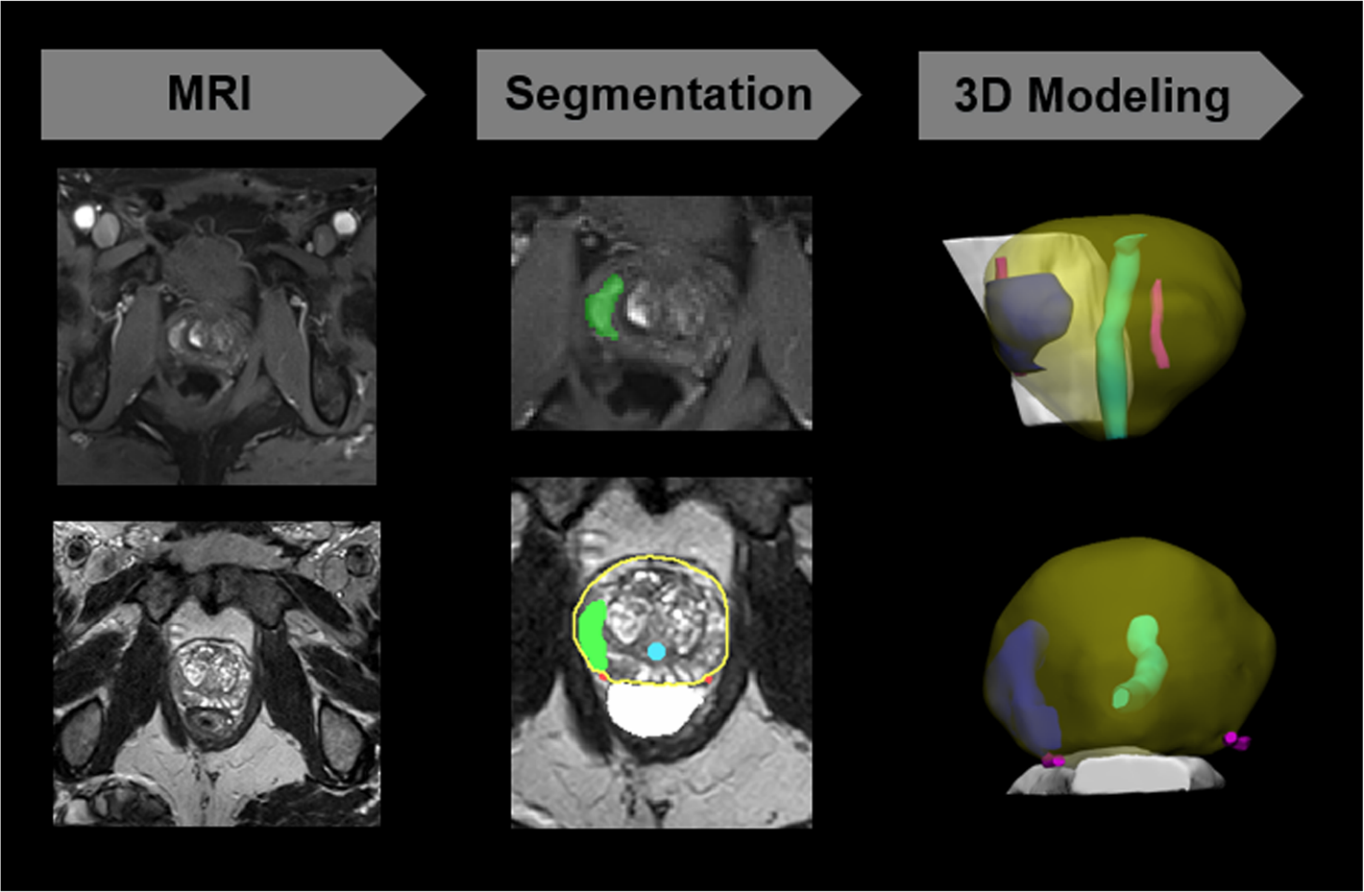
Example workflow for the creation of 3D prostate cancer models. Left: MRI with representative diffusion weighted and T2 SPACE images, Middle: Segmentation of the dominant lesion (green) on the DWI and tumor (green), prostate (yellow outline), neurovascular bundles (pink), urethra (turquoise), and rectum (white), and Right: 3D modeling of the prostate with antero-lateral and inferior views: prostate (yellow), lesion (blue), neurovascular bundles (pink), urethra (green), rectal wall (white)

Patient-specific 3D anatomical prostate cancer models that highlight the prostate, prostate tumor, urethra, neurovascular bundles, and rectal wall were created from the mpMRI data **(**Fig. 1**)** [33]. The T2-weighted spin-echo sequence with high sampling efficiency (SPACE) images were used for the primary segmentation, and if necessary, in order to well-visualize the lesion, diffusion-weighted imaging (DWI) or dynamic contrast-enhanced (DCE) sequences were co-registered to the SPACE series. Regions of interest were segmented by a single user with 16 years of medical imaging experience (NW) using a combination of manual and semi-automated methods (Mimics 21.0, Materialise, Leuven, BE). Volumes were automatically calculated based on the segmented region regions and segmented regions were visualized in 3D format with computer-aided design (CAD) software (3-matic, Materialise, Leuven, BE).

### Cryotherapy probes

**Fig. 2:**
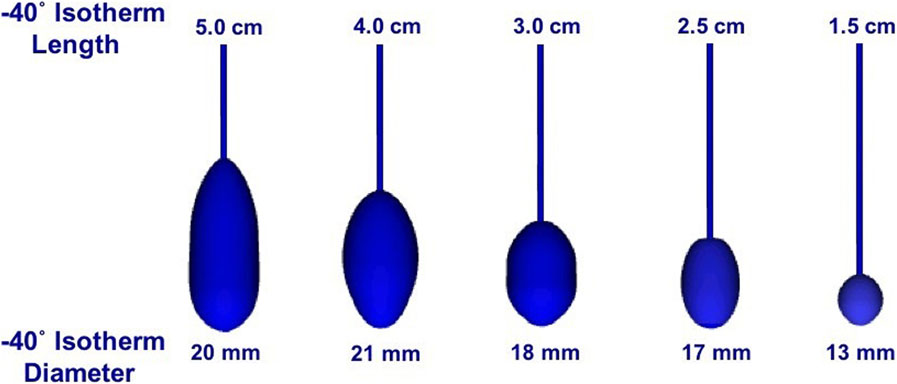
3D virtual cryotherapy probes of multiple dimensions to simulate different − 40 °C ice-ball dimensions

Virtual cryotherapy probes were designed by the first author (NW) using the 3-matic CAD software to emulate the − 40 °C isotherm volumes from published dimensions. Virtual − 40 °C isotherms were created for 1.5 cm, 2.5 cm, 3.0 cm, 4.0 cm, and 5.0 cm cryoprobe volumes **(**Fig. 2**)**.

### 3D procedure planning/simulations

Virtual treatment simulation was performed by two of the co-authors (NW and JSW) in the 3-matic software for all patients in the 3D planning group pre-treatment and retrospectively post-treatment for the 2D planning group. The 3D prostate model was oriented in a supine position allowing the simulation to be performed in the same alignment as the cPGA operating procedure and a 1 cm margin was created around each tumor. Virtual cryotherapy probes were then selected and manually placed into the software in a spatial orientation to ensure confluent − 40 °C isotherm encompassing both the tumor and the margin. This model was assessed in multiple views to ensure treatment confluence. The distances between the center of each probe were measured in order to reproduce the plan during the operation. In addition, contours of the anatomy and selected cryotherapy probes were generated on the 2D MR images.

### Operating procedure

All cryoablation procedures were performed by the planning surgeon (JSW) under general anesthesia in a dorsal lithotomy position. A BK Flexfocus 800 biplanar ultrasound probe (model # 8808) attached to a Civco brachytherapy stand and stepper was utilized to visualize the prostate. Healthtronics™ cryoablation equipment was utilized to perform all ablations procedures.

#### 2D planning method

For patients undergoing treatment with 2D planning, the Healthtronics™ software package was utilized to plan probe location. This software utilizes a 2D rigid registration of the prostate in an axial view on ultrasound. Probe placement is then guided by the 2D software in order to optimize probe-to-probe distance, probe-to-capsule distance, and probe -to urethra distance. This software does not utilize any MR-US fusion technology. MR tumor location is targeted using visual estimation. Visual estimation is performed preoperatively using image measurements on axial and sagittal MR images. These measurements are translated to real-time US imaging to achieve visual estimation in lesion targeting. Cryotherapy probes are then placed under axial and sagittal ultrasound guidance. Each needle is placed via a 16 gauge brachytherapy grid with 2.5 mm distance between each grid location.

#### 3D planning method

The same software and equipment as described above is utilized for 3D planning with the exception of the pre-treatment planning as described above. The location of the pre-planned cryoprobes are then placed according to the 3D treatment planning, also using visual estimation. Again, no fusion software was available on the ultrasound for these ablation procedures.

#### Cryoablation procedure

After completing cryoablation needles according to the treatment plan, thermocouples are placed into specific treatment locations in order to provide real-time temperature monitoring of critical locations including treatment margins and safety monitors. Cystoscopy is then performed to ensure that no needles traverse the urethra. Next, a urethral warming catheter is placed and the cryoablation cycle is initiated. Freezing proceeded from anterior needles to posterior glands. Propagation of the ice is monitored using ultrasound imaging in axial and sagittal views. Treatment efficacy is further assessed with real-time evaluation of thermocouple temperature to ensure achievement of target temperature in the treatment zone and to maintain sufficiently warm temperatures in critical regions such as the rectum and external sphincter. Two freeze-thaw cycles were performed, and the total freeze time and nadir temperatures were recorded. Operating times were also recorded for patients. A Students t-test was performed to determine if there was a difference between 2D and 3D planning groups (Matlab 2017a, The Mathworks Inc., Natick, MA). The number of cryotherapy probes planned was compared to the number utilized.

### Evaluation of treatment

In order to measure treatment efficacy, treatment zone biopsy results at 6 months were evaluated. Post-operative MRI and PSA at 3 and 6 months were also performed. The Kruskal-Wallis H Test was performed to determine if there was a difference in positive biopsy rates for the 2D and 3D planning groups. Statistical evaluation was carried out in SPSS Software (IBM, Armonk, NY).

## Results

Forty men successfully underwent cPGA. The median age of the cohort undergoing 3D treatment planning was 64.8 years with a median pretreatment PSA of 6.97 ng/mL. The Gleason grade group (GGG) of treated index lesions in this cohort included 1 (5%) GGG1, 11 (55%) GGG2, 7 (35%) GGG3, 1 (5%) GGG4. Two (10%) of these treatments were post-radiation salvage therapies. The retrospective 2D treatment cohort included 20 men with a median age of 68.5 years, median pretreatment PSA of 6.76 ng/mL. The Gleason grade group (GGG) of treated index lesions in this cohort included 3 (15%) GGG1, 8 (40%) GGG2, 8 (40%) GGG3, and 1 (5%) GGG4. Two (10%) of these treatments were post-radiation salvage therapies.

**Fig. 3:**
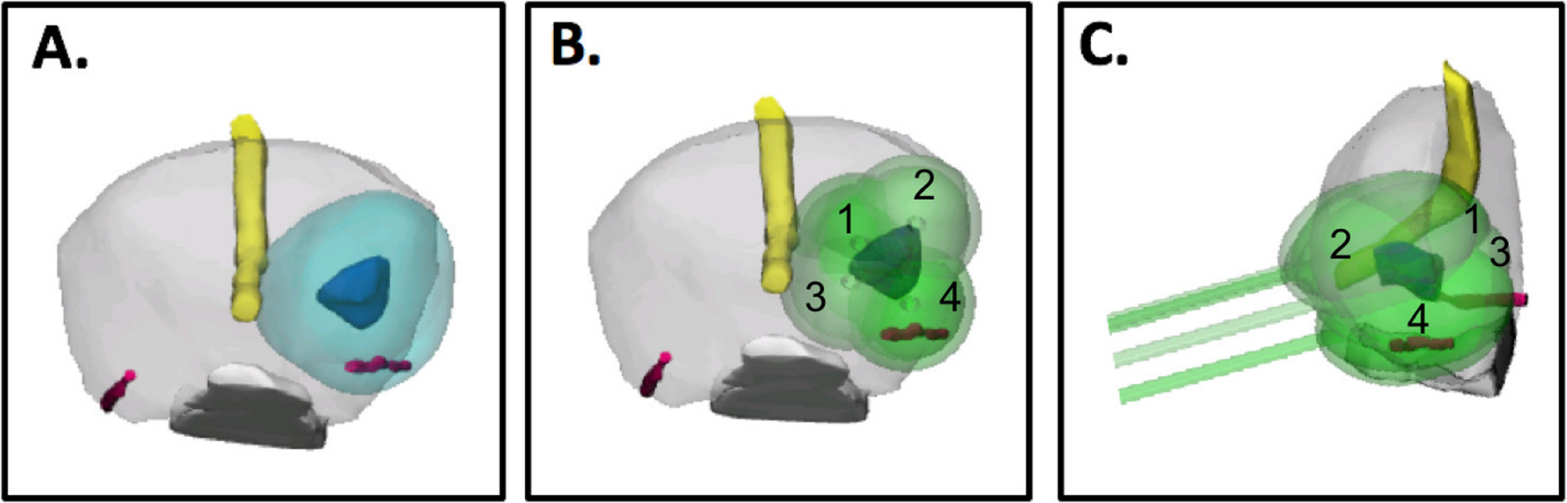
**a** 3D prostate cancer model viewed from the apex (prostate – light gray, urethra – yellow, neurovascular bundles – pink, rectal wall – white, tumor – dark blue, tumor with 1 cm margin – cyan). **b** 3D model from part a) shown with 4 cryotherapy probes (light green) placed over the tumor and 1 cm margin. **c** Sagittal view of model with probe placement. Note that in this view probes 1 and 2 are overlapping as are 3 and 4

**Fig. 4:**
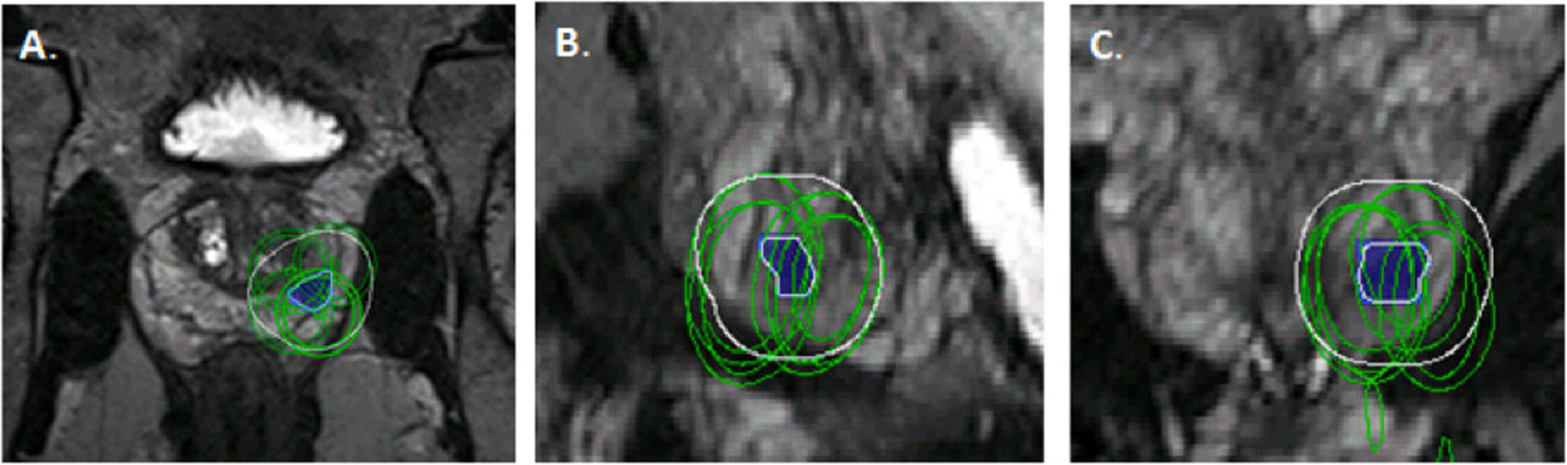
**a** Axial, **b** Sagittal, and **c** Coronal images from 3D T2-Weighted MR sequence with the lesion (blue), outline of the 1 cm margin (white), and outline of the cryotherapy probes (green)

The 3D surgical plan was successfully simulated prior to the procedure in all 40 patients: 20 patients prospectively selected to undergo pre-procedural 3D planning and 20 patients with retrospective 3D planning. 3D planning for a representative patient is shown in Fig. 3 and contours of this 3D plan are shown overlaid onto the 2D T2-Weighted MR images in Fig. 4. All patients in the 3D planning group successfully underwent the focal cryotherapy procedure following the 3D simulation. The number of cryotherapy probes utilized matched the plan in 16/20 patients (80%). For the four patients where the plan did not match the actual amount utilized, more cryoprobes were utilized in three cases and fewer cryobrobes were utilized in one case. Discrepancy in planned to utilized cryoprobes resulted from anatomical restrictions (gland size, inability of place needles as planned via grid). For the group with only 2D planning, the number of probes in the 3D plan matched the number utilized for 6/20 patients (30%), predicted that more probes should be utilized for 11/20 patients (55%), and predicted fewer probes for 3/20 patients (15%).

The number of cryoprobes predicted in the 3D plan was 3.89 ± 1.50 in the 3D group with prospective planning and 3.90 ± 0.91 in the 2D group with retrospective 3D planning (*p* = 0.72). The average number of cryoprobes utilized in the actual procedure was 4.10 ± 1.37 and 3.25 ± 0.44 for the groups with 3D pre-operative planning and only 2D planning respectively (*p* = 0.01). Operating times were recorded for 15 patients with retrospective 2D planning and 14 patients with prospective 3D planning. The mean operating times were 100.47 ± 24.30 and 100.64 ± 13.19 min for the 2D and 3D groups respectively (*p* > 0.05).

For the 3D planning group, 18 patients returned for follow-up. Targeted biopsy was not performed in four of these patients: two patients with undetectable PSA, one patient with MRI negative findings and PSA = 0.58, and one patient with negative hybrid PET/MRI. Of these four patients, the number of cryotherapy probes planned matched the number utilized in three cases and predicted less than were utilized in the fourth case. For the remaining 14 patients, biopsy results at 6 months were negative for 13 patients (92.9%). In the single positive case, the patient had a 450mm^3^ lesion with a Gleason Score = 6, and the number of cryoprobes planned matched the number utilized (*n* = 4). Post treatment MRI was available in all patients and demonstrated ablation zone completely encompassing pre-treatment MR lesion in 18/20 (90%).

Thirteen patients in the 2D planning group returned for follow-up targeted biopsy and ten (76.9%) had negative 6 month post ablation biopsy. For the remaining three patients (23.1%) who were positive in the ablation zone, one patient with Gleason score 3 + 3 in the medial margin and two with Gleason score 3 + 4 in the treatment zone. Of these patients, the predicted plan using 3D modeling matched the actual plan in one case (4 cryoprobes planned and utilized) and predicted more cyroprobes in two cases: one case predicted 5 cryoprobes but only three cryoprobes were utilized and the other predicted 4 cryoprobes but only 3 were utilized. Although 3 patients had positive findings post-operatively in the 2D planning group as compared to one patient in the 3D planning group, this did not reach statistical significance (*p* = 0.056). No post-surgical complications were reported for either group; and no additional complications were associated with the increased number of cryoprobes used in the 3D cohort.

## Discussion

Due to significant treatment toxicities associated with both radiation and radical prostatectomy, PGA for prostate cancer aims to achieve oncologic control while mitigating side effects by limiting treatment to only regions of known cancer and preserving normal surrounding tissue. Multiple technologies have been employed for focal therapy including high-intensity focused ultrasound (HIFU), cryotherapy, electroporation, radiofrequency ablation, and photodynamic therapy [34–38].While an organ sparing strategy is widely employed in multiple oncologic treatments including kidney and breast cancer, employing this approach for prostate cancer has been limited by challenges in precise determination of tumor location and volume within the prostate gland [39].

Multi-parametric MRI is increasingly utilized for detection, localization, and staging of prostate cancer and offers a potential tool for image guided PGA of prostate cancer [40, 41]. Despite this significant advance, achieving a confluent “kill zone” for MRI-guided PGA remains a significant challenge. In this study, we report the use of 3D prostate cancer models used in conjunction with mpMRI and advanced 3D visualization software methods to plan and simulate a theoretic zone of cryoablation for image-guided cryotherapy ablation of prostate cancer.

The pre-operative 3D prostate cancer models are helpful in planning the cryotherapy procedure. These 3D models easily conceptualize the location of the tumor within the prostate as well as provide guidance on the extent of the necessary treatment margin (in this study a 1 cm margin was utilized) to predict an adequate “kill” zone. The 3D models also provide a comprehensive understanding of the 3D surgical anatomy including an understanding of the relationship of surrounding critical structures to the proposed treatment zone and can provide surgeons with improved confidence that they planned the procedure correctly [42].

The virtual cryotherapy probes also allowed the exact “kill zone” to be predicted pre-operatively, thereby facilitating the operating procedure. The procedure was successfully carried out in all patients following the 3D virtual surgical planning procedure. In regard to the cryotherapy probe selection, there was a strong correlation between the planned number and the actual number used in the surgical procedure (80%), which suggests that the 3D surgical plan helped to guide the procedure. Although there was no difference in operating times between groups, less variation was seen in the 3D planning group. In addition, in this small cohort, a greater number of patients in the 3D planning group were negative for cancer post-operatively as compared to those in the 2D planning group, with 1/17 (5.9%) and 3/13 (23.1%) positive for cancer at follow-up biopsy for the 3D and 2D groups respectively. Properly planning the number and size of cryotherapy probes could potentially impact the number of cyroprobes utilized for each procedure. As these probes are disposable, accurate pre-treatment planning potentially decreases the total cost of the procedure by avoiding utilization of unnecessary cryoprobes.

This study had several limitations including the small patient population and retrospective comparison cohort. In addition, this study did not use MRI-ultrasound fusion as it is not available. Finally, the pre-operative 3D procedure plan was performed cognitively due to a current lack of technology to provide 3D planning on existing cryoablation software platforms and may be prone to error; however, it has been shown that there is no significant difference in MRI targeting between cognitive and fusion biopsy [30].

Herein, the fact that 3D planning predicted a greater number of cryoprobes than 2D planning and that there was a higher success rate in the 3D cohort suggests that 3D planning allows for a more comprehensive assessment of the coverage area needed for successful tumor ablation. Future studies with more patients will assess how this method of procedure simulation compares to traditional 2D planning with mpMRI and how it impacts long-term treatment efficacy. In addition, the impact of providing real time guidance immediately on the same screen that provides ultrasound guidance will be assessed and a multi-center study will be performed to determine the actual impact of 3D planning on cPGA.

## Conclusions

This study represents a preliminary exploration of a novel 3D treatment planning approach to cPGA of the prostate. The metric of the number of cryoprobes aims to assess the adequacy of treatment volume. 3D treatment planning more accurately estimates treatment volume and thus may predict a larger number of cryoprobes. Meaningful differences between 3D planning and traditional 2D planning were not possible in this study due to the small cohort and retrospective nature of the evaluation; however, the results encourage additional study in a larger cohort.

## Data Availability

The datasets used and/or analyzed during the current study are available from the corresponding author on reasonable request.
